# Drug use and treatment success among gang and non-gang members in El Salvador: a prospective cohort study

**DOI:** 10.1186/1747-597X-8-20

**Published:** 2013-06-04

**Authors:** Knowlton W Johnson, Stephen R Shamblen, Matthew W Courser, Linda Young, Melissa H Abadi, Thom Browne

**Affiliations:** 1 Pacific Institute for Research and Evaluation, Inc., 300 S. Fourth Street, Ste. 300, Louisville, KY 40208, USA; 2 Bureau for International Narcotics and Law Enforcement Affairs, U.S. Department of State, 2201 C Street NW, Washington, DC 20520, USA

**Keywords:** Gangs, El Salvador, Drug abuse treatment, Outcome evaluation

## Abstract

**Background:**

This article focuses on examining drug abuse treatment (DAT) in El Salvador highlighting gang vs. non-gang membership differences in drug use and treatment outcomes.

**Methods:**

Cross-sectional and prospective cohort designs were employed to examine the study aims. The 19 centers that met the study’s inclusion criteria of one year or less in planned treatment offered varying treatment services: individual, group, family, and vocational therapy, dual diagnosis treatment, psychological testing, 12-step program, and outreach and re-entry aftercare. Most directors describe their treatment approach as “spiritual.” Data were collected from 625 patients, directors, and staff from the 19 centers at baseline, of which 34 patients were former gang members. Seventy-two percent (72%) of the former patients (448) were re-interviewed six-months after leaving treatment and 48% were randomly tested for drug use.

**Results:**

Eighty-nine percent (89%) of the DAT patients at baseline were classified as heavy alcohol users and 40% were using illegal drugs, i.e., crack, marijuana, cocaine, tranquilizers, opiates, and amphetamines. There were large decreases after treatment in heavy alcohol and illegal drug use, crime, and gang related risk activities. Gang members reported illegal drug use, crime, and gang related risk activity more than non-gang members, yet only 5% of the study participants were gang members; further, positive change in treatment outcomes among gang members were the same or larger as compared to non-gang members.

**Conclusions:**

Alcohol use is the drug of choice among DAT patients in El Salvador with gang member patients having used illegal drugs more than non-gang members. The study shows that DAT centers successfully reduced the use of illegal drugs and alcohol among gang and non-gang members. Although our study could not include a control group, we believe that the DAT treatment centers in El Salvador contributed to producing this treatment success among former patients. These efforts should be continued and complemented by funding support from the Salvadoran government for DAT centers that obtain certification. In addition, tailored/alternative treatment modalities are needed for gang members in treatment for heavy drinking.

## Background

Illicit drug use has increased dramatically in the past decade in the developing countries of Central America including El Salvador with a concomitant rise in substance use [1-4]. Since the late 1990s, the El Salvador market for cocaine, as an example, has greatly expanded and crack is a growing problem [5]. Like many middle- and low-income countries, El Salvador has served as a staging point for illegal drugs like crack that are destined for the United States.

El Salvador, a country of 7.2 million people which is bordered by Guatemala and Honduras, can be considered a developing nation that is struggling with very high unemployment rates, slow recovery from natural disasters such as a 1998 hurricane that ravaged parts of the country, a series of earthquakes in 2001 that killed over 1,000 people and destroyed over a quarter-million homes, and floods that left thousands homeless in 2009 (http://www.state.gov/r/pa/ei/bgn/2033.htm). El Salvador also has a long history of social and political instability. Deterioration of democratic institutions in the 1970s led to armed conflict from 1980 to 1992, during which 75,000 are estimated to have died (http://www.state.gov/r/pa/ei/bgn/2033.htm).

One result of the economic and political context of El Salvador has been the proliferation of gangs operating in both urban and rural areas of the country and gang-related drug trafficking [6,7]. Many Salvadorans left the country during the civil war, fled to the United States, formed new gangs such as MS-13, or joined existing gangs such as M-18. As these U.S. gangs grew, they then expanded their operations into Central American countries [8]. After the war ended in 1992, many gang members who were operating in the United States and other Salvadorans either migrated back to El Salvador or were deported as part of U.S. Immigration and Customs Enforcement policies that sent immigrants with criminal convictions back to their native countries [8,9]. Ribando [8] argues that gang-deportees “exported” a Los Angeles gang culture to Central America and then expanded this culture by recruiting new members from local populations. Estimates of gang membership in El Salvador range between 25,000 and 50,000 [10,11] and gangs are believed to be responsible for 60% of all murders in El Salvador [8].

Gangs in El Salvador are associated with drug trafficking as well as the use of illicit drugs, even though some research has suggested that gangs try to discourage drug use [12]. A 2001 study of nearly 1,000 Salvadoran gang members, which is the most recent gang related scientific drug survey, found that more than a fourth of those under study consumed crack daily and nearly two-thirds had consumed crack in the last month [13]. A qualitative study by Dickson-Gomez and colleagues [12] came to similar conclusions. Further, there is concern that this increase in illegal drug use among gang members may result in more risky sexual practices associated with HIV/AIDS [12,14].

The last national survey of illegal drug use conducted in El Salvador (2004) estimated the past 30 day use of crack among residents of San Salvador to be greater than 1% [15]. More recent estimates prepared by El Salvador’s Corte Suprema De Justicia Instituto De Medicina Legal and reported by the World Life Expectancy website suggest that alcohol use remains a serious problem among the general population in El Salvador [16]. El Salvador is ranked first among 192 countries in the number of annual deaths related to alcohol use (includes disease, homicides, traffic accidents, etc.) [16]. The rate of 22.8 annual deaths per 100,000 people is remarkable when compared to rates for Brazil (4.8) and the United States (2.1). More recent data from the [17] estimates that 21.3 annual deaths of male Salvadorans per 100,000 people could be attributed to liver cirrhosis and that 5.16% of males above age 15 and 1.01% of females above age 15 suffered from diagnosable alcohol use disorders. We were unable to find any statistics relating to gang use of alcohol or its consequences.

Although drug and alcohol abuse has become a growing problem across El Salvador, the only government-funded residential treatment center is located in the National Psychiatric Hospital in San Salvador [14]. In response to the problem of drug and alcohol abuse across El Salvador, a number of small, grassroots or community-based drug abuse treatment (DAT) centers have been established across the country [14]. Most of these drug abuse treatment centers in El Salvador are supported by religious groups and operated by recovering addicts and/or untrained volunteers from the faith community [18]. As a result, the treatment approaches used by the Salvadoran DAT centers vary, but are generally faith or Bible-based [6]. Because these Centers are small and poorly funded, staff training is usually experiential and accomplished through on-the-job interaction with other staff and patients. Moreover, few DAT centers in Salvador have sufficient funds to provide a career track for staff or the level of training and support that is needed for the programs to be effective.

In order to increase the capacity of DAT centers to provide evidence-based treatment services, the Organization of American States (OAS), obtained funding in 2007 from the U.S. Department of States’ Bureau of International Narcotics Control and Law Enforcement Affairs (INL) to implement a 10-week DAT training for center staff. OAS joined with the Anti-drug Foundation of El Salvador (FUNDASALVA), a non-governmental organization specializing in drug use prevention and treatment, to implement this training. The OAS training [called “Comisión Interamericana para el Control del Abuso de Drogas (CICAD) Basic Training”] was modeled after the Substance Abuse and Mental Health Services Administration’s (SAMHSA) TAP 21 Addiction Counseling Competencies training program and consists of 10 modules and 160 hours of coursework [19]. It included a mechanism to develop a national system to certify addiction counselors. In conjunction with the CICAD Basic Training, INL commissioned the Pacific Institute for Research and Evaluation (PIRE) to conduct a process evaluation of the training in DAT centers across El Salvador that still employed staff receiving the training. PIRE was also asked to conduct an outcome evaluation of change in treatment outcome with attention given to differences between treatment success of gang and non-gang members. Only the outcome evaluation and its results are presented in this article.

## Methods

Despite the importance of the evaluation of drug abuse treatment (DAT) worldwide, we found no assessment of DAT treatment in El Salvador. Further, there have been no comparisons of gang and non-gang membership differences in DAT treatment success in El Salvador. As such, this study’s primary focus is to assess the prevalence and type of drug use of center patients in treatment and centers’ treatment success with attention focusing on gang and non-gang members in treatment. The specific aims are to assess the:

1. (a) Extent and type of drug and alcohol use, criminal activity, and gang related risk activity among patients who enter drug abuse treatment in El Salvador and (b) differences between gang and non-gang patients.

2. (a) Level of treatment success among former patients for drug abuse and related problems after leaving treatment and (b) differences in treatment success that exist between gang members in comparison to non-gang members. Treatment success is defined as the level of statistical significance change in outcomes from baseline to six-month follow-up following treatment.

We employed a cross-sectional design toexamine baseline data about the prevalence of illegal drug and heavy alcohol use of the entire sample as well as a comparison of use by gang and non-gang members (Aim 1a, b). Aims 2a and 2b concerned an assessment of change in treatment outcomes using a prospective cohort design over time from baseline to six-month follow-up. The study’s measures, center recruitment and selection, data requirements, and analysis strategy are described below.

### Study measures

#### Outcomes

Four types of outcomes were included in this study.

➣ *Prevalence of illegal drug use* includes measures of overall use and drug specific use 30-day period prior to treatment and in the six-month follow-up interview (0 = no use, 1 = yes);

➣ *Prevalence of heavy alcohol use* is defined as binge drinking (i.e., having five or more drinks at one sitting) one or more times 30 days prior to treatment and the six-month follow-up interview (0 = no, 1 = yes);

➣ *Prevalence of felony and misdemeanor crimes* are defined as crimes six months prior to the baseline and the six-month follow-up interview (0 = no felonies/misdemeanors, 1 = one or more felonies/misdemea-nors); and

➣ *Extent of gang related risk activity* is defined as a count of the occurrence of 11 possible activities commonly associated with gang membership. Initially, from a larger list of activities, 11 were identified by an in-country DAT expert panel (n = 5) from the field of drug abuse and corrections professionals in El Salvador as associated with gang related activities. They included presence of tattoos; carried a weapon for protection; had gang activity in their neighborhood; had a friend or relative that belonged to a gang; been invited by a gang member to join a gang; been forced to join a gang; participated in the initiation of a new gang member; had confrontations with gang members; had problems with a gang because of their drug use; been involved in the trade of drugs with gangs; or experienced gang activity in their neighborhood. Cronbach’s alpha and the average intercorrelation of these 11 activities were acceptable at baseline (α = 0.72, r = 0.49, respectively). These risk behaviors were measured for the time frame six months prior to treatment and the follow-up interview, regardless of the length of treatment.

#### Moderating variable

A third variable that affects the direction and/or strength of the relationship between an independent variable (e.g., changes due to drug abuse treatment) and an outcome (e.g., drug use).

➣ *Gang membership * is measured by having an experienced interviewer query each center director during weekly on-site visits to the centers to identify or re-affirm existing patients who were gang members (0 = no, 1 = yes).

#### Control variable

An Inverse Mills Ratio (IMR) described in the analysis section was created from the following patient characteristics to control for potential attrition bias:

➣ Born in El Salvador (0 = no, 1 = yes);

➣ Only lived in El Salvador (0 = no, 1 = yes);

➣ High school graduate (0 = no, 1 = yes);

➣ Lived with family prior to treatment (0 = no, 1 = yes);

➣ Lived with partner prior to treatment (0 = no; 1 = yes);

➣ Unemployment (0 = no, 1 = yes);

➣ age (18–76 years); and

➣ Peer antisocial behavior scale—big arguments or fights, illegal drug use, drug trafficking, other illegal acts, time with gangs, arrested (0 = never, 1 = rarely, 2 = sometimes, 3 = often) – α = 0.84.

### Center recruitment and selection

At the outset, drug abuse treatment (DAT) centers and their directors were identified and invited to participate in the study because they oversaw staff who attended an Organization for American States (OAS) substance abuse treatment training in 2008 and 2009, which is described in the research setting section. These centers presented a large range of organizations that operate DAT centers in El Salvador, although most were from small community-based DAT centers with some functioning as shelters.

A total of 44 organizations with DAT programs were sent a screener questionnaire by one of our in-country project partners in order to collect preliminary data that the project team would use to select the participating DAT centers. The screener survey instrument focused on center-level characteristics-not individual-level data-and includes questions related to center size, drop-out rates, staff capabilities, type of patients served, etc., but also willingness of the organization to participate in the study. After center closures, study refusals, and study criteria (inclusion of 12 months or less planned length of stay), 19 DAT centers representative of the types of DAT operating in El Salvador agreed to participate in the study (see [20] for a detailed description of these procedures). These 19 DAT centers were located the capital city of San Salvador, in nearby municipalities such as San Miguel and Ilobasco, and in the outer regions of the country. They provided community-based DAT services for patients and had a majority of staff participate in the OAS substance abuse treatment training described in the research setting section.

### Data requirements

Five full-time field interviewers completed in-depth training on the collection of data using the five instruments fielded for this study: (1) a Director’s Interview, (2) a Staff Training Interview, (3) a Staff Training Assessment Questionnaire, and (4) Patient Baseline and (5) Follow-up Interviews. First, for data relating to this article, field interviewers completed a 45-minute in-person interview with each of the participating 19 center directors. The directors’ interview provided descriptive data regarding organizational characteristics, financial support, and organizational functioning of participating DAT centers. In addition, 92 staff from the 19 centers provided demographic data in a larger self-administered training assessment questionnaire.

A sample of 625 patients was interviewed at baseline within one to seven days after completing detoxification and then again approximately six months after leaving treatment. Patients were invited to participate in the evaluation whether they had stayed only one week in treatment or up to 12 months. Written consent was required for both the baseline and six-month follow-up interview and the drug test described below. Four-hundred and forty-eight (448) former patients from 19 DAT centers participated in the follow-up interview yielding a 72% study retention rate.

Former patients were informed prior to the post-treatment interview that they may be randomly selected for drug testing with their consent. Urine testing of a random sample of 48% of the patients interviewed at follow-up (215) showed very high levels of consistency between the self-reported drug and the urine test results, suggesting that the self-reported drug use prevalence rates are reliable measures. Specifically, 95% of the patients showed consistency with their urine test results when reporting about cocaine (κ = .54), 97% showed consistency when reporting about benzodiazepine/tranquilizer use (κ = .19), and 99% showed consistency when reporting about marijuana (κ = .81).

Interview questionnaires, research methods, and procedures were reviewed and approved by the Institutional Review Board of the Pacific Institute for Research and Evaluation. A two-step informed consent process was employed where patients first consented to the release of their names and admission dates to the research team, and then to participate in the study. Consent forms and interview questions were read aloud by the interviewers to the patients due to low literacy levels. Patients were informed that their participation was voluntary, that their decision on whether to participate would not result in any penalties or benefits to them or the center, that their responses would be confidential, and that they could decline to answer any questions.

### Analysis strategy

Our analysis strategy consisted of cleaning/processing the data and the then we proceeded to (1) construct scales, indexes, and new single item measures, (2) conduct an attrition analysis, (3) conduct a missing value analysis, and (4) conduct descriptive/inferential analyses.

#### Construction of measures

Measures were constructed using either (1) multiple Likert-type items measuring the same construct for which we took the mean, (2) multiple dichotomous items for which we took the count of occurrences, or (3) single-item measures of whether a behavior occurred (e.g., prevalence of marijuana use). We calculated Cronbach’s alphas and performed principle component analyses (also referred to as exploratory factor analysis) to examine their psychometric properties and internal consistency, respectively. Measures representing the count of behaviors (4) typically represent emergent constructs, which do not conform to traditional psychometric theory (see [21]). This type of measure is sometimes referred to as an index.

#### Missing value analysis

Missing background characteristic data were imputed using the expectation maximization (EM) algorithm in SPSS 18.0. EM employs maximum-likelihood estimation to ensure consistency between the variance-covariance matrix derived from the observed data and the imputed data [22]. As the amount of missing data were minimal and due to the necessity of eliminating any case with any missing background characteristic, we felt that imputation posed fewer inferential risks than eliminating entire cases.

#### Attrition analysis

To partially correct for a potential attrition bias, a Heckman two-step procedure was implemented. These methods are not subject to the same biases that attend propensity methods [23,24]. The first step probit regression model regressed attrition status on patient background characteristics not directly and *causally* related to outcomes (i.e., age, native Salvadoran status, having lived in El Salvador one’s entire life, less than high school education, living with family prior to treatment, living with relationship partner prior to employment, and unemployment prior to treatment). There was little evidence to suggest that patient background characteristics were related to attrition, as the overall model failed to achieve statistical significance [χ^2^(617) = 625.59, p = .397]. Nevertheless, there was one significant predictor of attrition, where those living with their family prior to treatment were less likely to drop out of the study than those not living with their family (21% vs. 37%, z = −4.01, p < .001). It is important to note that there was minimal evidence of attrition biases; however, we chose the conservative option for step two by producing a predicted score from the model, transforming it to an inverse Mill’s ratio (IMR), and including it in all inferential analyses.

#### Descriptive and inferential analyses

For Aim 1a, we conducted a descriptive analysis of the prevalence of substance use, criminal behavior, and gang related risk activity prior to entering treatment by examining descriptive statistics consisting of frequencies and counts for each variable. Differences in baseline standing between gang and non-gang members (Aim 1b) were examined using hierarchical linear model (or mixed model regression) random intercept regressions. These models regressed baseline treatment outcomes on the IMR and a dummy variable representing gang membership (0 = non-gang membership and 1 = gang membership).

Three-level hierarchical linear models (or mixed model regressions) for continuous outcomes and three-level hierarchical non-linear models for dichotomous outcomes were produced to address Aims 2a (change in treatment outcomes) and 2b (relative change in treatment outcomes for gang and non-gang members). These models were run as random intercept regressions, where variability was modeled as arising due to repeated observations for some patients (level two) and observations being nested within treatment centers (level three). These models more conservatively and realistically adjusted effect estimates for these sources of variability, which can spuriously contribute to possible program effects [25]. Dealing with observations as being nested within patient also confers the benefit of being able to use all of the data, regardless of whether a patient has a full complement of repeated observations. This approach is consistent with an intent-to-treat approach, which reflects that we really do not know what happens to those who drop out of our study.

All analyses regressed each outcome on a coded contrast representing time and the inverse Mill’s ratio. We also performed these models regressing outcomes on time, length of treatment in days, the orthogonal interaction between these two variables, and the inverse Mill’s ratio. Length of treatment and the interaction between length of treatment and time were not significant predictors in any analysis and the effects found in the reported models persisted despite these additional statistical controls. Thus, variable length of time in treatment does not serve as a credible alternative explanation for our findings, so we only report the more parsimonious models. The models testing Aim 2b also included a contrast representing gang membership and the orthogonal interaction between time and gang membership.

## Results

### Profile results

As context for results that address the study’s aim, we provide first a profile of the patients, center directors, staff, and center structure and treatment services.

#### Patients

A total of 625 patients from the 19 centers participated in this study, of which 34 participants were identified as gang members. The ages of the sample ranged from 19 years old to 76 years old with a mean age of 38 (SD = 10). Only 20 of the 625 (3%) patients were female. About a fourth of the patients had lived in other countries besides El Salvador, most often in the U.S. Slightly less than a fourth of the patients (24.5%) had completed at least a high school education. About half of the patients (53.1%) had been living at home with relatives prior to treatment and nearly three-fourths (73.6%) were not married nor living with a companion. Over half were either not working (43.7%) or only working occasionally at odd jobs (16.6%).

The background profile of gang members differed slightly from the larger sample of patients. A total of 34 patients from 13 of the 19 centers were identified as gang members. The ages of the gang members ranged from 19 years old to 62 years old with a mean age of 30 (SD = 7). Of these 34 gang members, 100% were male. About one-fifth (21.2%) of the gang members had lived in other countries besides El Salvador. Slightly less than one-fifth of the gang members (18.2%) had completed at least a high school education. About half of the gang members (51.5%) had been living at home with relatives prior to treatment and about two-thirds (60.6%) were not married nor living with a companion. Over half of gang members were either not working (39.4%) or only working occasionally at odd jobs (15.2%).

#### Center directors and staff

All 19 DAT center directors and 92 staff participated in the study. The directors were mostly male (95%); and, on average, they chose the age category of 40 and 49 years old. More than a third (42%) had less than a high school education and approximately one fifth (21%) had completed college. Most directors (90%) indicated they were a recovering alcohol or drug addict and had worked in their centers for 8 to 10 years. The centers have an average of six full-time administrative and therapeutic staff (ranging from 2 to 30 across centers; SD = 6.0), and an average of four part-time staff (ranging from 0 to 11; SD = 2.9). Of the 92 study staff members, a large majority were male (88%), 30 years of age or older (98%), and were recovering alcoholics or drug addicts (80% with an average of 12 years being sober; SD = 8.0).

#### Center infrastructure and treatment capacity

Directors characterized their centers as residential treatment centers. All center directors indicated they offered individual and group therapy and 94% described their treatment approach as “spiritual.” Eighty-five percent of the centers indicated that they also offered family therapy, 68% offered vocational therapy, 58% offered psychological testing, re-entry aftercare, and dual diagnosis treatment. A little more than half (57%) stated they used a 12-step program and 45% reported offering outpatient services. The planned length of in-patient stay for the centers varied, with 11% having a 3-month length of stay, 6% having a 6-month length of stay, 44% having a 7- to 11-month length of stay, and 39% having a 12-month length of stay. On average, centers reported that 10% of patients dropped out before the first 30 days of treatment. The average actual length of stay in treatment was 51.2 days (SD = 56.0).

On average, the number of beds reported by the centers ranged from 8 to 550, with an average of 59 beds reported per center (SD = 120.7). Fifteen of the 19 centers reported serving only adult males (78%) and three centers stated they served only adult females (7%). Nine DAT centers reported that the majority of their patients were court-mandated (47%), eight reported serving dual-diagnosed patients (42%), seven DAT centers reported serving juveniles along with adults (37%), six reported serving patients with HIV/AIDS (32%), and six centers reported serving mentally ill patients (32%).

Adequate financial resources for operating the treatment centers are a challenge and funding tended to come from a variety of sources. Religious charity and individual contributions, selling products, and other fundraising activities were among the most commonly mentioned sources of funding. Eighteen of the 19 DAT center directors (95%) were full-time volunteers with only one being a full-time paid employee of the center. Only one center reported receiving financial support from government entities.

Record data from OAS indicated that of the 142 DAT center staff that participated in this INL funded program, 109 (77%) were still employed at the study centers at the start of data collection for this study. Of the 19 study directors, 14 (74%) attended the training.

Fourteen of the 19 center directors (73%) indicated their centers accepted gang members, including those mandated by the court. The two most common reasons given for why the remaining three DAT centers do not accept gang members included a lack of experience in treating gang members and the absence of gang members seeking treatment. Eleven of the 14 DAT center directors (79%) whose centers accept gang members reported that gang members live together with other patients in their DAT centers. The remaining three DAT center directors indicated that their centers separated gang members from other patients.

### Level of drug use, crime, and gang related risk activity: aim 1

**Figure 1:**
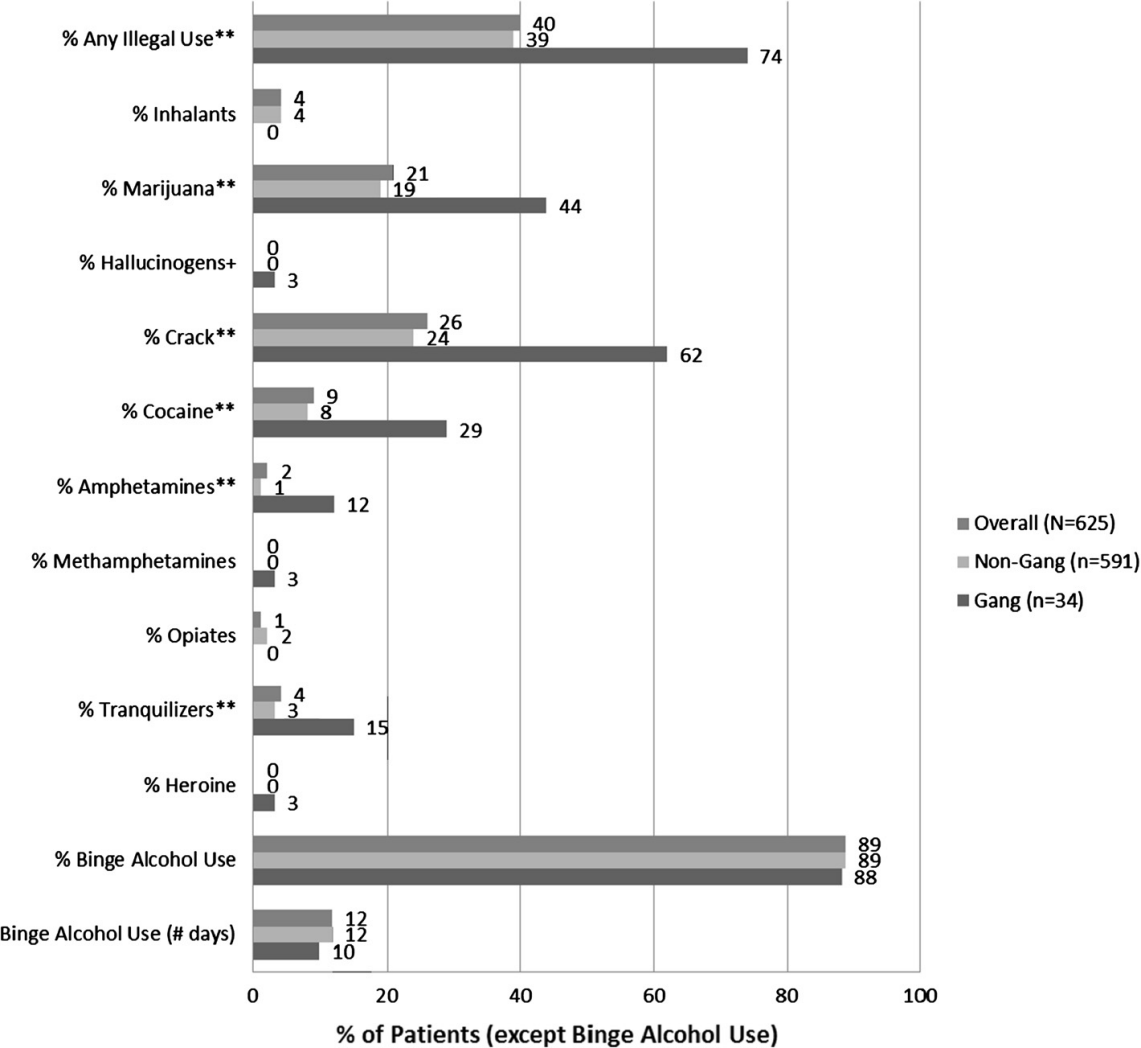
**Extent of drug and alcohol use of DAT patients and comparison of gang vs. non-gang activities in El Salvador.** Note: Statistical significance indicators indicate differences between gang and non-gang members. ** p < .01, * p < .05, + p < .10.

Figure 1 shows that 89% of the patients reported problem drinking as defined by one or more episodes of binge drinking in the past 30 days prior to treatment. On average, binge drinking occurred 11.7 days out of the past 30 days (SD = 10.9). Forty percent (40%) of the DAT patients reported use of any illegal drug in the past 30 days prior to treatment. The most prevalent use in descending order of prevalence proportions were: crack (26%), marijuana (21%), marijuana and crack (10%), cocaine (9%), marijuana and cocaine (5%), tranquilizers (4%), and inhalants (4%). The gang and non-gang member drinking pattern was similar with prevalence and frequency of binge drinking, which did not show evidence of a difference (all p ≥ .05). In contrast, gang members were much more likely than non-gang members to use any illegal drugs [74% vs. 39%, t(623) = 3.84, p < .001], marijuana [44% vs. 19%, t(623) = 3.38, p = .001], crack [62% vs. 24%, t(622) = 4.48, p < .001], cocaine [29% vs. 8%, t(623) = 2.39, p = .018], and tranquilizers [15% vs. 3%, t(623) = 3.42, p < .001].

**Figure 2:**
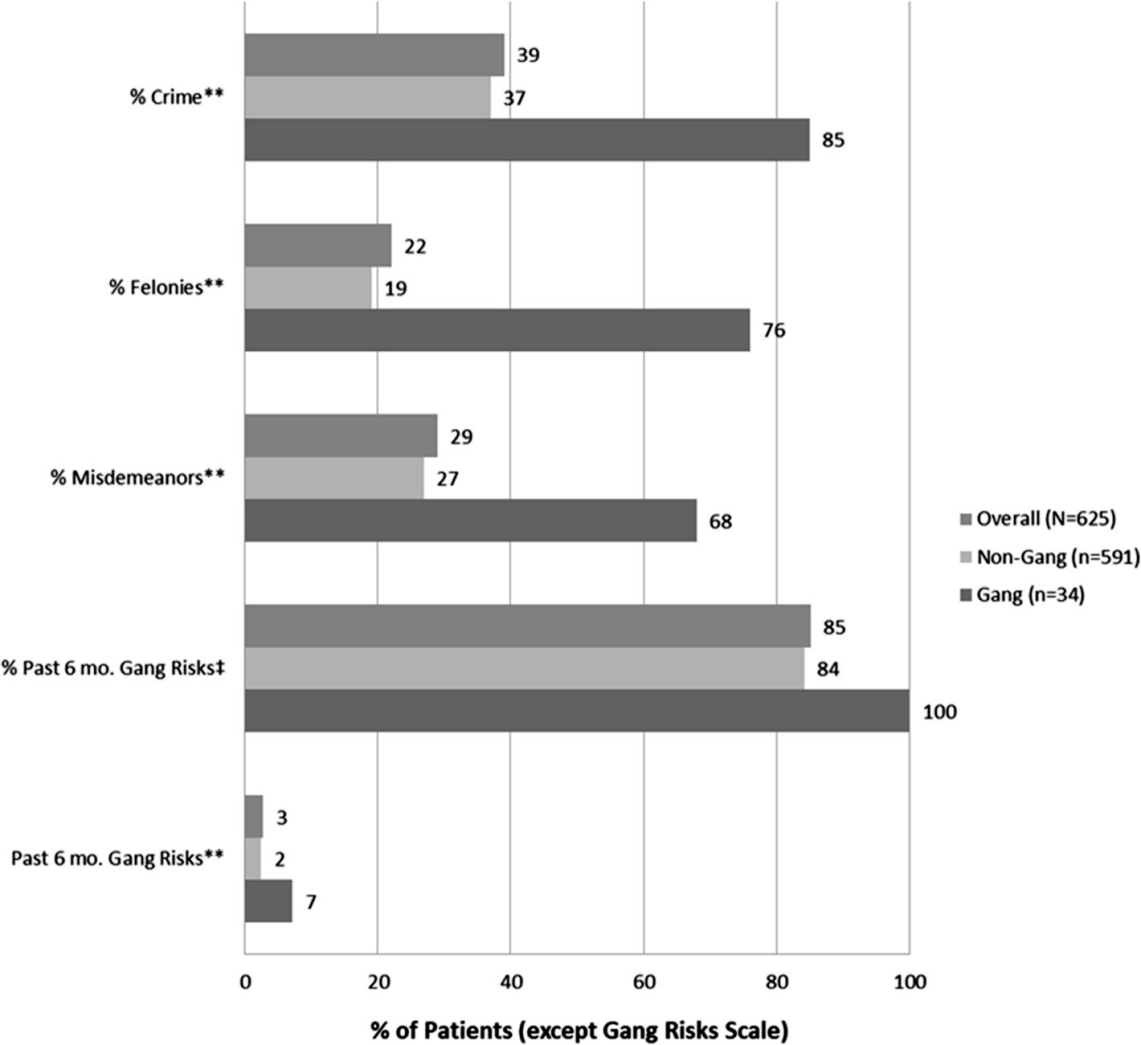
**Extent of crime and gang related risks of DAT patients and comparison of gang vs. non-gang activities in El Salvador.** Note: Statistical significance indicators indicate differences between gang and non-gang members. ** p < .01, * p < .05, + p < .10. ‡ Could not be compared, due to gang risks being constant for gang members.

Figure 2 shows that more than one-third (39%) of the patients indicated having engaged in either a felony (22%) or misdemeanor (29%). Further, they indicated having engaged on average, in 2.7 (SD = 2.2) gang related risk activities in the past six months. Not surprisingly, the differences between gang and non-gang members were large for any crime [85% vs. 37%, t(623) = 4.46, p < .001], felonies [76% vs. 19%, t(623) = 6.28, p < .001], misdemeanors [68% vs. 27%, t(623) = 4.26, p < .001], and past six-month gang risk activity [7.2 incidents (SD = 1.9) vs. 2.4 incidents (SD = 2.3), t(623) = 13.95, p < .001].

### Change in DAT outcomes: aim 2

Tables 1 and 2 present the effect sizes (odd ratio and correlation), test statistics, and level of significance for the four treatment outcomes. Additional baseline vs. six-month follow-up assessment percentage differences are presented in the corresponding text. Table 1 shows that changes speculated to be due to DAT activities exhibited medium-to-large decreases from baseline to the six-month follow-up assessment of any past month illegal substance use (40% vs. 10%) and number of days binge alcohol use in the past month [11.7 (SD = 10.9) vs. 3.1 (SD = 6.9) days]. Similarly, there were medium to large decreases in past month crime (39% vs. 10%) and past six-month gang related risk activity [2.7 (SD = 2.2) vs. 1.3 (SD = 1.7) activities].

**Table 1:** Changes in DAT outcomes

	**Past month any illegal substance use**		**Frequency of past month binge drinking**		**Past month crime**		**Past 6 mo. gang risk activity**	
	**t**	**OR**	**t**	**r**	**t**	**OR**	**t**	**r**
Intercept	1.32	2.05	7.83**	.88	1.51	2.17	8.59**	.90
Inv. Mills	−2.23*	.39	−1.76+	-.07	−2.59*	.35	−2.52*	-.10
Time	−10.26**	.15	−15.72**	-.43	−9.84**	.17	−15.16**	-.42
	χ^2^	ICC	χ^2^	ICC	χ^2^	ICC	χ^2^	ICC
Repeated Observation Variance	675.22*	.16	718.18**	.08	632.54	.08	1543.42**	.47
Center Variance	53.44**	.08	47.04**	.03	45.79**	.05	19.86	.00

** p < .01, * p < .05, + p < .10; N = 625; t-tests are based on 18 df for the intercept, 623 df for the inverse Mill’s ratio, and 1070 df for time; effect size r calculated using the formula reported in Cohen [26]: r = [ t^2^ / ( t^2^ + df ) ]^.5^.

**Table 2:** Changes in DAT outcomes by gang membership

	**Past month any illegal substance use**		**Frequency of past month binge drinking**		**Past month crime**		**Past 6 mo. gang risk activity**	
	**t**	**OR**	**t**	**r**	**t**	**OR**	**t**	**r**
Intercept	1.65	2.99	5.83**	.81	2.60*	5.68	11.20**	.94
Inv. Mills	−2.32*	.37	−1.75+	-.07	−2.70**	.33	−2.91**	-.12
Gang Membership	3.20**	3.53	-.12	.00	3.11**	3.76	11.98**	.43
Time	−6.21**	.10	−5.56**	-.17	−6.55**	.07	−13.06**	-.37
Interaction	−1.11	.67	1.55	.05	−2.41*	.37	−6.77**	-.20
	χ^2^	ICC	χ^2^	ICC	χ^2^	ICC	χ^2^	ICC
Repeated Observation Variance	664.56*	.16	723.99**	.08	625.12	.09	1319.41**	.40
Center Variance	57.18**	.09	46.92**	.03	43.39**	.05	24.90	.01

** p < .01, * p < .05, + p < .10; N = 625; t-tests are based on 18 df for the intercept, 622 df for the inverse Mill’s ratio and the gang membership main effect, and 1068 df for time and the interaction; effect size r calculated using the formula reported in Cohen [26]: r = [ t^2^ / ( t^2^ + df ) ]^.5^.

Table 2 presents results from a moderating or subgroup analysis of treatment success by gang vs. non-gang patients. These results show that while the treatment positively impacted both gang and non-gang members, success among gang members was higher in comparison to non-gang members for crime (non-gang: 37% vs. 9% and gang: 85% vs. 14%) and gang related risk activities [non-gang: 2.4 (SD = 1.9) vs. 1.2 (SD = 1.5) and gang: 7.2 (SD = 2.3) vs. 3.1 (SD = 2.9)]. Treatment impact on gang and non-gang members was about the same for 30-day prevalence of illegal substance use (non-gang: 39% vs. 9%, and gang: 74% vs. 18%) or past 30-day binge drinking frequency [non-gang: 11.8 (SD = 10.9) vs. 3.0 (SD = 6.1), and gang: 9.8 (SD = 10.2) vs. 4.8 (SD = 8.7) days].^a^

## Discussion

This study has produced results that profile patients, Center directors, and staff of the study. We also describe center structure, treatment services, and center policy about acceptance of gang members into treatment. These results are intended to provide context for outcome results relating to the extent and type of drug use, treatment success and outcome differences between gang and non-gang members. Of particular importance is that the study results show DAT center resources are limited with most of the center directors and staff being volunteers. The results also show that most El Salvadoran DAT centers accept gang members into treatment. However, police powers have been expanded by establishing a national law (Super Mano Dura) that allows the police to search and arrest suspected gang members regardless of whether they have committed a crime [27]. This law may be a potential deterrent to gang members entering treatment for drug and alcohol problems.

Alcohol is the drug of choice in El Salvador with problem alcohol use by gang and non-gang members. Interestingly, there are no differences between gang and non-gang members in problem alcohol use. In contrast, gang members in treatment use illegal drugs significantly more than non-gang members. These results are the first gang and non-gang treatment comparisons of problem alcohol use published for El Salvador.

DAT centers in El Salvador have been successful in treating both gang and non-gang members. These outcome results show large, positive, statistically significant, changes in alcohol and drug use, crime, and gang risk activity for both gang and non-gang members. These results also suggest that the gang members in El Salvador who enter DAT treatment benefit more from the existing combination of treatment strategies than non-gang members do as it pertains to criminal behavior, but not as it pertains to substance use. We were unable to find any evaluation in the literature that assessed treatment impact on substance abuse among gang members in comparison to non-gang members.

While there has been no research that has compared substance abuse treatment for gang and non-gang members, there is a consistent body of research on the past 30 years that supports the effectiveness of drug treatment in general in the U.S. and other countries (e.g., [28,29]) and residential drug abuse treatment (DAT) programs in particular [30-35]. It should be noted that these studies funded by the National Institute on Drug Abuse (NIDA) and other funding agencies are dated in some cases; and, none used a randomized controlled design although the treatment effects were medium to large in all studies.

### Policy implications

Several important policy implications of this study for developing countries need to be highlighted.

*Alternative forms of treatment should be explored for the El Salvador drug user treatment system*. We found that DAT centers in El Salvador use multiple treatment modalities that support the published DAT research that no single treatment is appropriate for all individuals. Increased treatment success might occur if the treatment setting, interventions, and services were matched to each individual’s particular problems and needs as recommended in NIDA’s *Principles of Drug Addiction Treatment*: *A Research*-*Based Guide*[36]. In addition to incorporating multiple combinations of therapies, research also shows that individual patients may require varying combinations of services (e.g., counseling, family therapy, medical). The El Salvadoran drug use treatment system may consider incorporating into its DAT model a continuing care approach to treatment by continually assessing and modifying, as necessary, individual treatment plans for each patient to ensure that it meets his or her changing needs [19].

Implementation of follow-up services after treatment for heavy drinking may assist gang members who have a drinking problem. Currently, DAT centers in El Salvador do not incorporate follow-up activities as part of their center mission. This phase of treatment may increase treatment success even more than reported in this study. For example, in Peru and Brazil, some drug user treatment programs among street children incorporate a follow-up strategy that may increase treatment success [37].

In our study, we found that while the existing DAT centers with varying combinations of treatment strategies show medium to large reductions of illegal drug use among both non-gang and gang members, these treatment strategies only show small reductions of heavy drinking among gang members. While we could not find any evidence-based alcohol related treatment program for gang members, it is clear that gang members with alcohol abuse problems have to be treated differently.

*National policy should promote allocation of national*-*level funding to DAT centers that meet existing national certification standards established by the National Drug Commission with support of OAS*-*CICAD*. *To date*, *85 addictions counselors have been certified by the Commission*. The study shows that resources for DAT centers are sparse with a large majority of the center operating with volunteer directors and staff and there are limited funds that have to be raised by each center to cover operational cost. Some governmental funding for establishing and/or maintaining evidence-based treatment and a certification system is paramount to sustainable drug abuse treatment in El Salvador.

*National policymakers in developing countries like El Salvador should advocate for a national system of collecting*, *analyzing*, *and disseminating results from DAT data for both the extent and type of drug use prior to entering treatment and change in these outcomes*. Directors and staff need to know if alcohol is the drug of choice of DAT patients (as is the case in El Salvador) as well as the frequency and type of illegal drugs being used. Knowing the extent of treatment success is also important. This information can be helpful in providing the most effective delivery to those in treatment. The Therapeutic Community drug abuse treatment model has a data collection and analysis component, but it is often a weak component of the model [37]. Although national governments or DAT programs in developing countries may not have resources to collect and analyze such data, program directors should reach out to a local university to assist them as part of students’ research training.

### Study limitations

The treatment study in El Salvador has several limitations that should be noted. First, the response rate of 72%, is less than response rates reported for many treatment evaluation studies conducted in the United States [38]. However, we feel this is an acceptable rate given the challenges of conducting treatment evaluation in developing countries. Tracking methods, like the use of social security numbers, are non-existent; communications infrastructure is rudimentary; poor transportation and road systems inhibit travel; and access to and security in the high-crime communities where many addicts live is lacking. Further, limited DAT center resources make it difficult for centers to support baseline data collection and implement a satisfactory tracking system. We did conduct a selectivity analysis to determine predictors of attrition from 625 to 448 (72% vs. 28%) study participants, and then constructed an attrition bias correction covariate for all longitudinal analyses. Interestingly, the treatment attrition between non-gang and gang members was similar.

Second, we do not have a control group to increase our confidence in the treatment effects results produced, although a prospective cohort group design has been acceptable in drug user treatment research in the past. Further, since treatment success was measured by reductions in the prevalence of illegal drug use, frequency of heavy alcohol use, crime, and gang related risk activities was proven to be substantial, we believe that the DAT treatment centers in El Salvador contributed to producing this treatment success. Third, these results can only be generalized to DAT centers participating in the El Salvador evaluation, or future community based DAT centers that are similar to those in the study. However, these study results do have policy implications of DAT programs that have a planned treatment longer than 12 months or which are being implemented in correctional facilities. Fourth, we also would be remiss if we failed to note that results about the DAT center implementation quality is missing in this study. This was intentional in that this research focused on outcomes, not on implementation of the treatment per se.

## Conclusions

Our evaluation of 19 community-based drug treatment centers in El Salvador found that alcohol use is the drug of choice among DAT patients in El Salvador and that gang members in treatment reported having used illegal drugs more frequently than non-gang members. The study shows that DAT centers successfully reduced the use of illegal drugs and alcohol among both gang and non-gang members. Although our study could not include a control group, we believe that the DAT treatment centers in El Salvador contributed to producing this treatment success among former patients. These efforts should be continued and complemented by funding support from the Salvadoran government for DAT centers that obtain certification. In addition, tailored/alternative treatment modalities are needed for gang members in treatment for heavy drinking. Further research should focus on empirically documenting the treatment models used by community-based DAT centers in El Salvador, along with the impact of contextual changes in funding, training, and certification on DAT center operations and outcomes.

## Endnote

^a^It could be argued that these results are biased due to our small sample size of gang members (n = 34 at pre-test and n = 22 at post-test). More specifically, gang members (35%) had a higher proportion of study attrition than non-gang members (28%), which could mean gang members were more likely to drop out of the program to return to substance use or gang membership, leaving gang members more likely to have positive outcomes to be included in our analysis. To examine a worst case scenario where both gang and non-gang members dropping out of the study exhibited negative behavior, we performed a variant of last observation carried forward imputation [39], assuming negative outcomes for those who dropped out of the study. Statistical significance decisions were not changed for any of our gang membership X time interactions in this worst case scenario analysis, suggesting gang member attrition is not a plausible explanation for the reported results.

## Abbreviations

CICAD: Inter-american drug abuse control commission; CSAT: Center for substance abuse treatment; DARP: Drug abuse reporting program; DAT: Drug abuse treatment; DATOS: Drug abuse treatment outcome studies; EM: Expectation maximization; FUNDASALVA: Anti-drug foundation of El Salvador; IMR: Inverse mills ratio; INL: International narcotics and law enforcement; NIDA: National institute on drug abuse; NTORS: National treatment outcome research study; OAS: Organization for American States; PGC: Project guidance committee; SAMHSA: Substance abuse and mental health services administration; TOPS: Treatment outcome prospective study

## Competing interests

There are no financial competing interests relating to this research.

## Authors’ contributions

KJ directed the preparation of the manuscript and prepared text for Results and Discussion and Conclusions and Policy Implications; MC directed the fieldwork, assisted in preparing the results section, and conducted content editing; SS prepared the data, conducted the HGLM analysis, and prepared the Results text, figures, and tables; LY contributed to the background section, conducted literature searches, and content editing; MA prepared the References section and conducted content editing; and, TB provided inserts to the Conclusions and Policy Implications and conducted content editing. All authors read and approved the final manuscript.

## References

[B1] Obot IS Limits of substance-use interventions in developing countries Lancet 2007 369 1323 1329 10.1016/S0140-6736(07)60373-0 17448801

[B2] Perngparn U Assanangkamoachai S Pilley C Aramratanna A Drug and alcohol services in middle-income countries Curr Opin Psychiatry 2008 21 229 233 10.1097/YCO.0b013e3282fc1ea3 18382219

[B3] Thirtalli J Chand PK The implications of medication development in the treatment of substance use disorders in developing countries Curr Opin Psychiatry 2009 22 3 274 280 10.1097/YCO.0b013e32832a1dc0 19346946

[B4] Uchtenhagen A Substance use problems in developing countries Bull World Health Organ 2004 82 9 641 15628199 PMC2622977

[B5] Dickson-Gomez J Bodnar G Guevara CE Rodriguez K Rivas De Mendoza L Corbett AM With God’s help I can do it: crack users’ formal and informal recovery experiences in El Salvador Subst Use Misuse 2011 46 426 439 10.3109/10826084.2010.495762 20735191 PMC3704222

[B6] Dickson-Gomez J Structural factors influencing patterns of drug selling and use and HIV risk in the San Salvador metropolitan area Med Anthropology Quarterly 2010 24 157 181 10.1111/j.1548-1387.2010.01095.x PMC3721422 20550091

[B7] Dickson-Gomez J Factores Estructurales relacionados a las drogas y violencia en El Salvador. El impacto de las drogas en la violencia: buscando soluciones 2004 El Salvador: United Nations Development Program

[B8] Ribando C Gangs in Central America. CRS Report for Congress 2005 Retrieved April 20, 2011 from http://fpc.state.gov/documents/organization/47140.pdf

[B9] Reisman L Breaking the vicious cycle: responding to central American youth gang violence School of Advanced Int Stud Rev 2006 26 147 152

[B10] Archibold RC New York Times. Gangs’ truce buys El Salvador a tenuous peace 2012 Retrieved November 24, 2012 from http://www.nytimes.com/2012/08/28/world/americas/in-el-salvador-gang-truce-brings-tenuous-peace.html?emc=eta1

[B11] Bremer C El Salvador gangs big headache for new patient [Electronic Version]. Reuters 2009 Retrieved July 5, 2010 from http://www.reuters.com/article/2009/03/17/idUSN17291185

[B12] Dickson-Gomez J Bodnar G Guevara A Rodriguez K Gaborit M HIV risk among street gangs in El Salvador J Gang Res 2006 13 2 1 26

[B13] Santacruz Giralt M Concha-Eastman A Barrio adentro: La solidaridad violenta de las pandillas 2001 San Salvador, El Salvador: OAS, Instituto Universitario de Opinion Publica

[B14] Dickson-Gomez J McAuliffe T Rivas de Mendoza L Glasman L Gaborit M The relationship between community structural characteristics, the context of crack use, and HIV risk behaviors in San Salvador, El Salvador Subst Use Misuse 2012 47 265 277 10.3109/10826084.2011.635325 22217125 PMC3263344

[B15] Carr D Bodnar G Guevara A Rodriguez K Guevara R Estudio Nacional: Prevalencia del consume de substancias psicoactivas en El Salvador 2004 San Salvador: Fundacion Antidrogas de El Salvador (FUNDASALVA)

[B16] WorldLifeExpectency.com Health profile: El Salvador 2012 Retrieved May 21, 2013 from http://www.worldlifeexpectancy.com/country-health-profile/el-salvador

[B17] World Health Organization (WHO) El Salvador: Socioeconomic context 2011 Retrieved June 7, 2012: http://www.who.int/substance_abuse/publications/global_alcohol_report/profiles/slv.pdf

[B18] CICAD & OAS Training and certification for drug treatment counselors makes its mark in El Salvador [Electronic Version] 2008 Retrieved July 5, 2010 from http://www.cicad.oas.org/oid/NEW/Information/Observer/08_02/default.asp

[B19] SAMHSA [Substance Abuse and Mental Health Services Administration] Substance abuse: Clinical issues in intensive outpatient treatment Center for Substance Abuse Treatment. Treatment Improvement Protocol (TIP) Series, No. 47, Report No.: (SMA) 06–4182 2006 Rockville (MD) Author 22514853

[B20] Courser M Johnson K Young L Shamblen S Vanderhoff J Carr D An outcome evaluation of drug treatment in El Salvador. Research Monograph 2011 Louisville, KY: Pacific Institute for Research and Evaluation-Louisville Center

[B21] Bollen KA Lennox R Conventional wisdom on measurement: a structural equation perspective Psychol Bull 1991 10 305 314

[B22] Dempster A Laird N Rubin D Maximum likelihood from incomplete data via the EM algorithm J R Stat Soc, Series B 1977 39 1 38

[B23] Heckman JJ The common structure of statistical models of truncation, sample selection and limited dependent variables and a simple estimator for such models Ann Econ Soc Meas 1976 5 475 492

[B24] Heckman JJ Sample selection bias as a specification error Econometrica 1979 47 153 161 10.2307/1912352

[B25] Raudenbush SW Bryk A Hierarchical Linear Models 2002 2 Thousand Oaks, CA: Sage

[B26] Cohen J Statistical power analysis for the behavioral sciences 1988 2 New York: Academic Press

[B27] Ribando-Seelke C Gangs in Central America, CRS Report for Congress 2011 Retrieved January 3, 2011 from http://assets.opencrs.com/rpts/RL34112_20110103.pdf

[B28] Gossop M The clinical fallacy and treatment outcomes Addiction 2008 103 89 90 18081613 10.1111/j.1360-0443.2007.02093.x

[B29] Simpson DD Curry SJ Special issue: drug abuse treatment outcome study (DATOS) Psychol Addict Behav 1997 11 211 337

[B30] Condelli WS Hubbard RL Relationship between time spent in treatment and patient outcomes from therapeutic communities J Subst Abuse Treat 1994 11 25 33 10.1016/0740-5472(94)90061-2 8201630

[B31] Hubbard EL Craddock SG Flynn PM Anderson J Etheridge RM Overview of 1-year follow-up outcomes in the drug abuse treatment outcome study (DATOS) Psychol Addict Behav 1997 11 261 278

[B32] Johnson KW Young L Pan T Zimmerman RS Vanderhoff KJ Therapeutic Communities (TC) drug treatment success in Thailand: A 2006 follow-up study. Research Monograph 2007 Louisville, KY: Pacific Institute for Research and Evaluation-Louisville Center

[B33] Johnson K Pan Z Young L Vanderhoff J Shamblen SR Browne T Therapeutic community drug treatment success in Peru: a follow-up outcome study Subst Abuse Treat Prev Policy 2008 3 1 15 10.1186/1747-597X-3-1 19055774 PMC2631528

[B34] Johnson K Young L Shamblen SR Suresh G Browne T Chookhare W Evaluation of the therapeutic community treatment model in Thailand: policy implications for compulsory and prison-based treatment Subst Use Misuse 2012 47 889 909 10.3109/10826084.2012.663279 22676561

[B35] Melnick G Hawke J Wexler HK Client perceptions of prison-based therapeutic community drug treatment programs Prison J 2004 84 121 138 10.1177/0032885503262459

[B36] National Institute on Drug Abuse Principles of drug addiction treatment: A research-based guide 2012 3 National Institutes of Health, U.S. Department of Health and Human Services: NIH Publication No. 12-4180

[B37] Johnson KW Young LC Suresh G Berbaum ML Drug abuse treatment training in Peru: a social policy experiment Eval Rev 2002 26 480 519 10.1177/019384102236521 12243105

[B38] Scott C A replicable model for achieving over 90% follow-up rates in longitudinal studies of substance abusers Drug Alcohol Depend 2004 74 1 21 36 10.1016/j.drugalcdep.2003.11.007 15072804 PMC5937263

[B39] Ali MW Talukder E Analysis of longitudinal binary data with missing data due to dropouts J Biopharm Stat 2005 15 993 1007 10.1080/10543400500266692 16279357

